# Correlation Between Perfusion Abnormalities Extent in Ventilation/Perfusion SPECT/CT with Hemodynamic Parameters in Patients with Chronic Thromboembolic Pulmonary Hypertension

**DOI:** 10.4274/mirt.galenos.2020.31932

**Published:** 2021-02-09

**Authors:** Salih Özgüven, Selin Kesim, Kevser Öksüzoğlu, Mehmed Yanartaş, Serpil Taş, Feyza Şen, Tunç Öneş, Sabahat İnanır, Halil Turgut Turoğlu, Bülent Mutlu, Tanju Yusuf Erdil, Bedrettin Yıldızeli

**Affiliations:** 1 Marmara University Pendik Training and Research Hospital, Clinic of Nuclear Medicine, İstanbul, Turkey; 2 University of Health Sciences Turkey, Kartal Koşuyolu Training and Research Hospital, Clinic of Cardiovascular Surgery, İstanbul, Turkey; 3 Marmara University Pendik Training and Research Hospital, Clinic of Cardiology, İstanbul, Turkey; 4 Marmara University Pendik Training and Research Hospital, Clinic of Thoracic Surgery, İstanbul, Turkey

**Keywords:** Chronic thromboembolic pulmonary hypertension, ventilation/perfusion scintigraphy, mean pulmonary arterial pressure, pulmonary vascular resistance, 6-minute walk distance

## Abstract

**Objectives:**

Chronic thromboembolic pulmonary hypertension (CTEPH) is a type of pulmonary hypertension with persistent pulmonary vascular obstruction and exercise intolerance, which may benefit from pulmonary endarterectomy (PEA). Ventilation/perfusion (V/Q) scan is the preferred screening test of CTEPH, which can be used to assess the anatomical extent of the disease. This study aimed to analyze the correlation between the extent of mismatched Q defects in V/Q single photon emission computed tomography/computed tomography (SPECT/CT) with preoperative clinical and hemodynamic parameters in patients with CTEPH.

**Methods:**

A total of 102 patients with CTEPH prior to PEA having V/Q SPECT/CT scans were retrospectively reviewed. Age, gender, New York Heart Association classification, intraoperative right-sided heart catheterization (mPAP and PVR), and 6-minute walk test (6MWT) findings were obtained from clinical records of patients.

**Results:**

Linear regression analysis showed a significant but weak correlation between the preoperative mPAP and PVR with the extent of mismatched Q defects in V/Q SPECT/CT (rs=0.09474 with p=0.0016 and rs=0.045 with p=0.045, respectively). No significant correlation was found between 6MWT and extent of mismatched Q defects in V/Q SPECT/CT (p>0.05).

**Conclusion:**

A quantitative assessment of Q defects on V/Q SPECT/CT might provide information about hemodynamic parameters in patients with CTEPH.

## Abstract (tr)

**Amaç:**

Kronik tromboembolik pulmoner hipertansiyon (KTEPH), kalıcı pulmoner vasküler obstrüksiyon ve egzersiz intoleransı ile karakterize bir pulmoner hipertansiyon grubudur ve pulmoner endarterektomiden (PEA) fayda görebilir. Ventilasyon/perfüzyon (V/Q) sintigrafisi, hastalığın anatomik yaygınlığını değerlendirmek için kullanılabilen KTEPH’nin tercih edilen tarama testidir. Bu çalışmanın amacı, KTEPH tanılı hastalarda ameliyat öncesi klinik ve hemodinamik parametreler ile V/Q tek foton emisyon tomografisi/bilgisayarlı tomografideki (SPECT/BT) Q defektlerinin yaygınlığı arasındaki korelasyonu araştırmaktır.

**Yöntem:**

PEA ile tanısı doğrulanmış 102 KTEPH hastasının PEA öncesi V/Q SPECT/BT görüntüleri retrospektif olarak incelendi. PEA öncesi yaş, cinsiyet, New York Kalp Derneği sınıfı, intraoperatif sağ kalp kateterizasyonu sonuçları [ortalama pulmoner arter basıncı (OPAB), pulmoner vasküler rezistans (PVR)] ve 6 dakikalık yürüme testi (6DYT) gibi klinik parametreleri hastaların klinik kayıtlarından elde edildi.

**Bulgular:**

Doğrusal regresyon analizi, preoperative OPAB ve PVR ile V/Q SPECT/BT’deki eşleşmeyen Q defektlerinin yaygınlığı ile anlamlı ancak zayıf bir korelasyon gösterdi (rs=0,09474, p=0,0016; rs=0,045, p=0,045). 6DYT mesafesi ile eşleşmeyen Q defektlerinin yaygınlığı arasında ise anlamlı bir ilişki bulunmadı (p>0,05).

**Sonuç:**

V/Q SPECT/BT’de saptanan Q defektlerinin kantitatif değerlendirilmesi, KTEPH tanılı hastalarda hemodinamik parametreler hakkında fikir sağlayabilir.

## Introduction

Chronic thromboembolic pulmonary hypertension (CTEPH) is a progressive pre-capillary pulmonary hypertension, which results from incomplete resolution of a pulmonary embolus, leading to elevated pulmonary vascular resistance (PVR), mean pulmonary artery pressure (mPAP), and right-sided heart failure (1,2). Acute embolism can vary from a total resolution to persistent perfusion (Q) defects after an adequate anticoagulation therapy. Approximately, 30% of patients have permanent defects after 6 months of anticoagulation; however, only 10% of defects consequently developed CTEPH (3).

CTEPH should be questioned in patients with abnormal ventilation (V)/Q scintigraphy including at least one mismatched segmental Q defect and imaging findings of organized thrombi in pulmonary arteries following >3 months of therapeutic anticoagulation (3). Invasive pulmonary angiography historically remains as the objective reference standard for diagnosis and evaluation for chronic emboli extent, whereas V/Q scan is the preferred first-line screening test for CTEPH (4,5). V/Q scintigraphy is used to diagnose and assess the anatomical extension of mold, and estimate therapy response in patients with CTEPH (6).

The only curative treatment option for CTEPH is the pulmonary endarterectomy (PEA) in appropriate patients (7). This technique is associated with improved survival, functional capacity, and quality of life (1,8). PEA may be related with high mortality rates regarding to the extent of the disease (9,10).

Factors that need to be assessed before PEA include the anatomical location and distribution of disease and left and right ventricular systolic functions (6). Previous studies showed that hemodynamic parameters play a crucial part in the evaluation of prognosis, disease severity, and operability (11,12). This study aimed to assess the association of the extent of mismatched Q abnormalities in V/Q single photon emission computed tomography/computed tomography (SPECT/CT) with preoperative hemodynamic and clinical parameters in patients with CTEPH.

## Materials and Methods

### Study Subjects

Over a period of nine years (January 2011 to May 2020), a total of 677 patients with a diagnosis of CTEPH at the preoperative evaluation underwent PEA. Of which, 102 patients with CTEPH whose V/Q SPECT/CT images obtained in our clinic prior to PEA were retrospectively reviewed.

Study exclusion incudes patients with isolated pulmonary artery vasculitis (n=8), hydatid cyst (n=8), pulmonary artery sarcomas (n=9), no mismatched V/Q defects in scintigraphy (n=7), and whose preoperative V/Q scans not acquired in our institution or lacking in our database (n=543).

Age, gender, New York Heart Association (NYHA) classification, intraoperative right-sided heart catheterization (RHC) (mPAP and PVR), and 6-minute walk test (6MWT) findings were obtained from clinical records of patients.

An informed consent was taken from all patients before the examination. Marmara University Faculty of Medicine Clinical Studies Ethics Committee approval was also obtained (date: September 2020, no: 09.2020.852).

### V/Q SPECT/CT Protocol

V/Q scans were performed with a one-day protocol (13). The V SPECT images were obtained before the Q scan. A 12-15 millicurie (444-555 megabecquerel) technetium‑99m (Tc-99m)-Technegas generated by the “TechnegasPlus” generator device (Cyclomedica Australia Pty Ltd., Australia) was used for the V phase. SPECT images of patients using a 180° dual head detector on SPECT/CT (Siemens Symbia TruePoint, Siemens Medical Solutions, USA) were acquired. Afterward, a Q SPECT with low dose CT scans was immediately obtained on the same table. After a slow (within 20-30 s) injection of 4-5 millicurie (148-185 megabecquerel). Tc-99m-macro aggregated albumin, (TechneScan LyoMAA; Mallinckrodt Medical) containing 100,000-200,000 particles, SPECT/CT was taken on the same device using similar SPECT parameters as those used for the V phase (low-energy high-resolution collimator, 128x128 matrix, 64 projections of 10 s, 1.00 zoom factor, and 140±10% keV energy window) and 13-25 mAs, 130 kV, and slice width of 5 mm for CT. Raw data of SPECT images processed with the “Tomo Reconstruction v.8.2.26.4” (Syngo‑Siemens AG) application and reconstruction was conducted with ordered subset expectation maximization method.

### Image Analysis

V/Q SPECT/CT images were evaluated by two nuclear medicine physicians without knowledge of the preoperative hemodynamic parameters and 6MWT records of patients. To figure out CTEPH severity, V/Q images were analyzed together for each pulmonary segment (14). Two physicians discussed each case to reach a final consensus.

### Clinical Data Analysis

RHC protocol and 6MWT protocol was carried out in accordance with previously described standard procedures (15,16,17). Data of the RHC (mPAP and PVR) and 6MWT were obtained from the clinical records of patients.

### Statistical Analysis

For continuous variables a mean value ± standard deviation and for categorical variables number and percentage were calculated. Per-segment basis analysis for each patient was used to estimate disease severity. Linear regression analysis was conducted to examine the concordance of mismatched Q defects severity on V/Q SPECT/CT with RHC and 6MWT results. Data analysis and graphs were plotted using GraphPad Prism version 8.0 for macOS, GraphPad Software, La Jolla California USA. P values of 0.05 or less were regarded as significant.

## Results

A statistical analysis of 102 patients with a diagnosis of CTEPH is presented, wherein 46 patients (45.1%) were women and 56 patients (54.9%) were men. The mean age of patients was 51.66 years (range of 19-77 years and standard deviation of 15.95). A total of 11 patients (10.8%) were NYHA class II, 74 patients (72.5%) were NYHA class III, and 17 patients (16.6%) were NYHA class IV. The mean preoperative mPAP and mean preoperative PVR were 43.73±14.77 mmHg and 681.62±411.75 dyn•s•cm^-5^, respectively. The mean 6MWT distance was 334.40±113.62 meters. The average number of abnormally perfused segments was 12.84±5.30.

**Figure 1:**
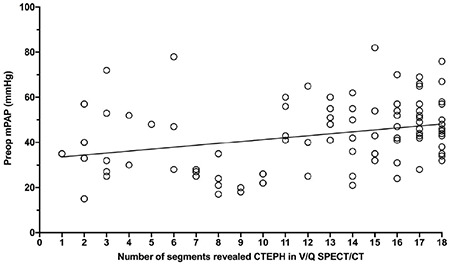
Correlation between preoperative mean pulmonary arterial pressure (mPAP) levels and number of segments revealed in chronic thromboembolic pulmonary hypertension ventilation/perfusion single photon emission computed tomography/computed tomography (V/Q SPECT/CT). Application of linear regression analysis revealed a significant but weak correlation between the preoperative mPAP and extent of mismatched perfusion defects in V/Q SPECT/CT (rs=0.09474 and p=0.0016) CTEPH: Chronic thromboembolic pulmonary hypertension, mPAP: Mean pulmonary arterial pressure, V/Q: Ventilation/perfusion, SPECT/CT: Single photon emission computed tomography/computed tomography

**Figure 2:**
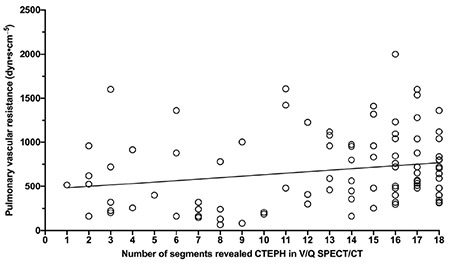
Correlation between preoperative pulmonary vascular resistance (PVR) and number of segments revealed in chronic thromboembolic pulmonary hypertension ventilation/perfusion (V/Q) single photon emission computed tomography/computed tomography (SPECT/CT). Linear regression analysis showed a significant but weak correlation between the preoperative PVR and extent of mismatched perfusion defects in V/Q SPECT/CT (rs=0.045 and p=0.045) CTEPH: Chronic thromboembolic pulmonary hypertension, V/Q: Ventilation/perfusion, SPECT/CT: Single photon emission computed tomography/computed tomography

The linear regression analysis showed a significant but weak correlation between the preoperative mPAP and PVR with the extent of mismatched Q defects in V/Q SPECT/CT (rs=0.09474 with p=0.0016 and rs=0.045 with p=0.045, respectively) (Figure 1, 2).

**Figure 3:**
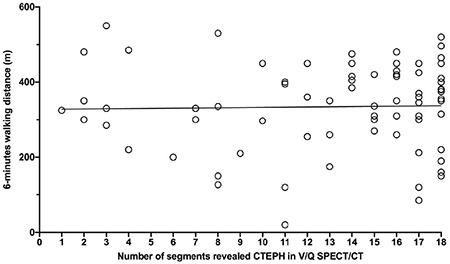
Correlation between 6-minutes walking distance and number of segments revealed in chronic thromboembolic pulmonary hypertension ventilation/perfusion (V/Q) single photon emission computed tomography/computed tomography (SPECT/CT). No significant correlation was found between 6-minute walk distance and extent of mismatched perfusion defects in V/Q SPECT/CT (p>0.05) CTEPH: Chronic thromboembolic pulmonary hypertension, V/Q: Ventilation/perfusion, SPECT/CT: Single photon emission computed tomography/computed tomography

No significant correlation was found between 6MWT distance and extent of the mismatched Q defects in V/Q SPECT/CT (p>0.05) (Figure 3).

## Discussion

The correlation of the degree of persistent thromboembolic disease (mismatched Q defects in V/Q SPECT/CT) with preoperative clinical and hemodynamic parameters was examined in the present research with the greatest number of patients with CTEPH proven by post-PEA surgical histopathological examinations. Results showed a statistically significant correlation between the number of mismatched Q defects in V/Q SPECT/CT and preoperative mPAP and PVR, but not with 6MWT. PVR and mPAP are the essential hemodynamic parameters in patients with CTEPH. Increased PVR is mainly caused by endothelial dysfunction, vasoconstriction, vascular remodeling, and obstruction of small pulmonary arteries. Interleukin-1 (IL-1), IL-6, and tumor necrosis factor-a are pro-inflammatory cytokines that are relevant to the pathogenesis (18). Dartevelle et al. (19) reported higher mortality rates for patients with PVR >900 dyn•s•cm^-5^ than those with PVR <900 dyn•s•cm^-5^. Furthermore, no patients with PVR <300 dyn•s•cm^-5^ pre-operatively died after PEA in a study performed by Yıldızeli et al. (17). In addition, increased mPAP, which induces right ventricular dysfunction, is found to be associated with higher mortality (20). In a study by Saouti et al. (21), the risk of mortality is higher in patients with mPAP >40 mmHg than those with an mPAP <40 mmHg. The association between the extent of the disease and hemodynamic parameters has been described in literature (22). Fukuchi et al. (23) found a correlation between planar Q index with mPAP and right ventricular ejection fraction using planar pulmonary Q scintigraphy. Recently, Derlin and colleagues, who investigated the correlation between V/Q SPECT/CT imaging findings and RHC, showed a statistically significant association between Q defect score, perfused lung volume, Q index with mPAP, and PVR (24). In line with other studies, a statistically significant difference between mismatched Q defects in V/Q SPECT/CT and preoperative mPAP (rs=0.095 and p=0.0016) and PVR (rs=0.045 and p=0.035) values was observed.

Patients with CTEPH generally display a decreased exercise capacity that is most commonly assessed with 6MWT. The prognostic value of the 6MWT has been reported in several studies (15,21). In a study by Reesink et al. (25), 6MWT had significantly increased one year after PEA, reflecting clinical and hemodynamic improvement (25). However, the correlation of the 6MWT distances with the extent of disease in CTEPH has not been widely studied. In fact, the 6MWT distances did not correlate with the number of mismatched Q abnormalities in our study. Variance in walking distance can be explained by the individual’s determinants such as age, sex, height, and weight on 6MWT.

### Study Limitations

Following are the limitations of this study. First, this study was designed as a retrospective, single-center study. Nevertheless, our study has the largest number of patients whose diagnoses were proven by histopathology. Second, it is not rare to find matched V/Q abnormalities in patients with CTEPH that are seen late in the course of the disease. Hence, this problem might lead us to underestimate the extent of disease-related defects.

## Conclusion

In conclusion, our study suggests that the extent of chronic thromboembolic disease revealed on V/Q SPECT/CT correlates with the preoperative hemodynamic parameters, thus predicting the severity and prognosis of the disease. Conversely, 6MWT was not found as a reliable indicator for the extent of the disease. Further studies are required in extended patient series to better represent the association between V/Q SPECT/CT Q defects with hemodynamic parameters and 6MWT in patients with CTEPH.
